# Translation and Cross-Cultural Adaptation of the Questionnaire on Stress in Diabetic Patients (QSD-R) to Brazilian Portuguese

**DOI:** 10.3390/healthcare12141375

**Published:** 2024-07-10

**Authors:** Amanda Vitória Zorzi Segalla, Silmara Meneguin, Carlos Antonio Negrato, Cesar de Oliveira

**Affiliations:** 1 Department of Nursing, Botucatu Medical School, São Paulo State University, São Paulo 18618-970, SP, Brazil; amanda.segalla@unesp.br (A.V.Z.S.); s.meneguin@unesp.br (S.M.); 2 School of Medicine, University of São Paulo, Bauru 17012-901, SP, Brazil; carlosnegrato@uol.com.br; 3 Department of Epidemiology & Public Health, University College London, London WC1E 6BT, UK

**Keywords:** diabetes mellitus, quality of life, surveys and questionnaires, psychological stress

## Abstract

Background: Diabetes mellitus is a chronic disease that can cause psychological stress. This study was conducted to perform the translation and cross-cultural adaptation of the QSD-R for diabetic patients into Portuguese (Brazil). Methods: This study was a cross-cultural adaptation process carried out in a public university in São Paulo, Brazil, in three stages: translation and backtranslation by two native-speaking independent professionals, analysis by a committee of specialists, and a pre-test phase. Results: The final version was created by seven experts after making semantic, idiomatic, and cultural changes to eighteen items. The results indicated a satisfactory content validation index (CVI ≥ 0.80). This version was applied to 30 patients. No item was excluded from the instrument. Conclusion: The translated version of the QSD-R was considered adequate and culturally adapted for use in Brazil to enable the psychometric validation of the instrument.

## 1. Introduction

Diabetes mellitus (DM) is a chronic metabolic disease that affects 347 million people globally [1,2,3]. It is estimated that by 2045, approximately 629 million people will be affected by this disease. Brazil is the fourth country with the highest number of cases, with a prevalence of 12.5 million people and a significant increase in the last 35 years [4]. This disease is directly related to the occurrence of cardiovascular diseases (CVDs), the risk of which increases progressively with the increase in fasting plasma glucose levels, even before reaching levels sufficient for diagnosis, and this is the main cause of death in this population [5,6].

Diabetic individuals, without previous CVDs, have a two to three times higher risk of vessel-related death compared to non-diabetic individuals, regardless of differences in age, sex, smoking status, and body mass index (BMI) [6]. Furthermore, microvascular complications such as retinopathies, nephropathies, and neuropathies are common in individuals with the disease and contribute to the increase in comorbidities [7].

Capillary blood glucose measurements constitute the essential pillar in the treatment of diabetes. Blood sugar assessments can be challenging due to various factors such as patient adherence issues, incomplete data, and irregular records with only a few readings taken throughout the day. Patients and/or family members also tend to forget to bring their blood glucose logs during medical appointments, which can further complicate the process [8]. Education for the self-management of diabetes is commonly provided in Brazil during outpatient appointments or at primary care units, but patients require ongoing support to maintain adherence [9].

Establishing a strong bond between healthcare providers and patients is crucial for ensuring the patient’s adherence to the therapeutic plan to achieve good metabolic control. This is especially important considering the challenges patients face in their daily lives due to disease management and physical or intellectual limitations [10].

However, difficulties concerning lifestyle changes can have unpleasant manifestations in the daily lives of patients [10]. They can affect their self-image, self-esteem, and role in the family/society, contributing to the emergence of depressive symptoms in the presence of physical limitations [11] and exerting a negative impact on quality of life (QoL) [12]. This impact depends on the perceptions of the patient and family, how they deal with self-care and the management of the disease, and the family dynamics as a whole [13].

Over the years, numerous studies have analyzed individuals with diabetes mellitus for complications and comorbidities, demonstrating the negative impact on quality of life [11]. One study assessed the psychological impact of the disease on QoL to gain a better understanding of the capacity of affected individuals to deal with a complicated, demanding treatment regimen. The authors found that 43% of the participants reported a severe impact on QoL and 48% reported severe impacts related to complications of the disease, such as skin alterations, as well as social and physical functioning [14].

In this population with DM, stress prevalence is high, especially among female patients, as predicted by various factors. Diabetes-related anguish is a syndrome with multiple symptoms, such as anxiety, conflict, frustration, and confusion. Psychological stress is present in many chronic diseases and it is being recognized as a risk factor for the onset and progression of a physical illness [15]. Stress is considered an important cause of health problems; however, this terminology has various meanings in research. In this study, they are defined as objective events or circumstances in everyday life, traumatic or not, that lead to the body’s stressful response [16]. Biologically, psychosocial stress can result in the chronic activation of the physiological response to stress, preventing the release of glucose and lipids into the circulation, the activation of the immune system, and the increase in blood pressure [16]. Furthermore, psychosocial stress can negatively impact self-care for type 2 diabetes, resulting in poor glycemic control [15,17].

A study conducted in Thailand with 77 diabetic women to investigate the effect of self-management education on the reduction in blood glucose, stress, and QoL found significant changes (*p* < 0.05) in HbA1c levels and levels of stress and QoL after the intervention [18].

Intending to measure stress in type I and type II diabetes, German researchers developed the Questionnaire on Stress in Diabetic Patients (QSD-R)—Revised in 1996. The final English-language version has forty-five items distributed among eight domains: leisure time, depression, hypoglycemia, self-medication, physical complaints, work, partner, and doctor–patient relationship. Reliability measured using Cronbach’s alpha coefficient ranged from 0.69 to 0.81. Each statement describes the impact on daily living and is scored on a scale of 0 (does not apply) to 5 (a big problem). The total score ranges from 0 to 225 points [19].

Considering the absence of measures available in Brazil to assess stress in patients with diabetes, the translation and cross-cultural adaptation of the QSD-R to Brazilian Portuguese can provide a reliable and reproducible scale for the collection and analysis of data on the stress experienced by individuals with diabetes and thus contribute to the reorganization of care practices.

Therefore, the present study aimed to perform the translation and cross-cultural adaptation of the QSD-R for diabetic patients into Portuguese (Brazil).

## 2. Methods

This study was conducted to translate and adapt the QSD-R to Brazilian Portuguese [19] following internationally recognized guidelines [20]. Cross-sectional data were collected from January to June 2023.

1. Translation by two independent translators whose main language is that to which the instrument will be translated and who are fluent in the original language of the instrument (English).
2. Synthesis of the translation in partnership between the translators and researchers in charge of this study. The goal of this phase is to finalize the new version of the instrument in the language proposed for this study.
3. Backtranslation by two translators fluent in the language to which the instrument was translated and the language of the original instrument (English). These translators must have no prior knowledge of the original document to avoid being influenced during the translation process. Backtranslation is important for determining whether the translated version reflects the same content as the original version, corresponding to a type of validity verification that enables highlighting gross inconsistencies or conceptual errors in the translation [20]. Through an analysis of the study’s authors and translators, a single synthesis version of the backtranslation into English is obtained for the subsequent assessment and considerations of the authors of the original instrument and composition of the final version.
4. Expert panel: The function of this step is to analyze the semantic, idiomatic, conceptual, and empirical language parameters of all versions for the subsequent definition of the final version to be administered to a small sample of patients who compose the pilot study [20].

The criteria adapted from Fehring [21] were used to select the specialists to compose the committee. According to the author, to be considered an expert in a specialization, the individual must have a doctoral or master’s degree with dissertations or theses relevant to the topic of interest, a paper published on the topic, and at least one year of clinical experience.

The experts received instructions on the objectives of this step, along with the versions of the questionnaire. A Likert scale from 1 to 4 was used: 1 = item not representative; 2 = item needs considerable revision to be representative; 3 = item needs some revision to be representative; and 4 = relevant or representative item. Three categories were assessed for all items on the scale and their respective response options: semantic/idiomatic, conceptual, and cultural [22].

Data analysis

The variables were analyzed descriptively. From these assessments, the content validity index (CVI) was calculated considering item responses with scores of 3 and 4. The CVI of each item corresponded to the sum of the number of 3 and 4 scores divided by the total number of responses. A minimum CVI of 0.80 was established for an item to be considered adequate [23]. The panel had the autonomy to alter, preserve, or eliminate items considered ambiguous or irrelevant. The members of the panel used the scores for all versions of the QSD-R scale [19].

5. Pre-test: 30 patients were invited to answer the pre-final version of the instrument [20]. The purpose of semantic analysis is to assess whether all items are adequate and understandable to the population for which the instrument was designed, and it should be administered to 30 to 40 individuals [20]. At this stage, patients with diabetes mellitus, over 18 years of age, literate, and who consented to participate in this phase of the research were included. Patients with visual difficulties were excluded.

**Figure 1:**
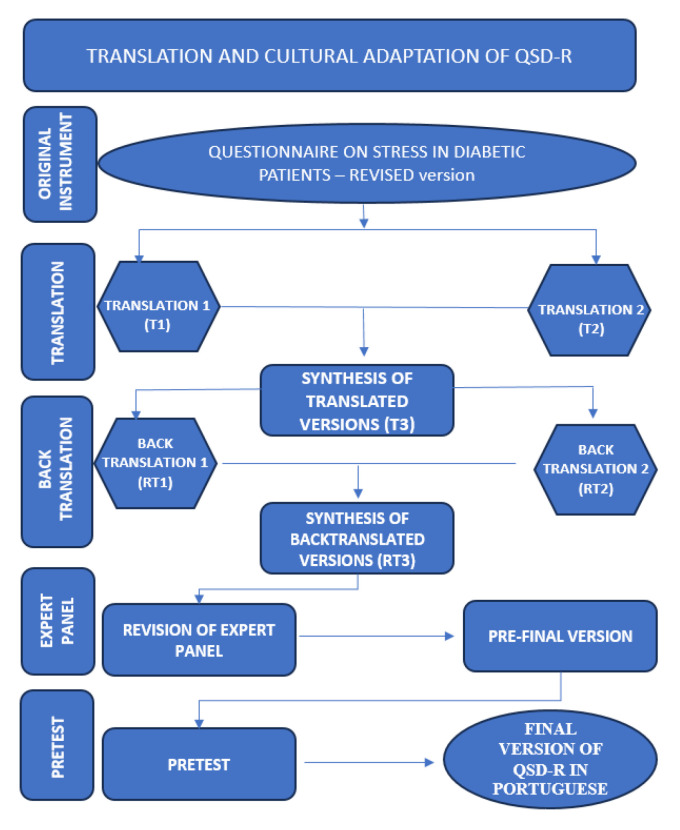
Flow diagram of the translation and cross-cultural adaptation of the QSD-R to Brazilian Portuguese, Botucatu, SP, Brazil, 2023. Source: the authors, 2023.

Each item was assessed based on adequacy/understanding: “Yes”, “No”, or “Partly”. An item was considered validated when achieving a minimum agreement rate of 75% positive answers. Items with a lower level of agreement were considered and analyzed for the possibility of being altered [24]. All steps of the translation and cross-cultural translation process are displayed in Figure 1. After the analysis of the expert panel and the pre-test, the developers of the original Questionnaire on Stress in Diabetic Patients (QSD-R)—Revised were contacted for consolidation of the final version.

This study received authorization from the authors of the original QSD-R, as well as approval from the Human Research Ethics Committee at São Paulo State University (UNESP), Sao Paulo (presentation certificate number: 1405.333.924; approval certificate number: 56981522.0.0000.5411).

## 3. Results

The translation and cross-cultural adaptation of the QSD-R to Brazilian Portuguese were performed by two native Brazilian Portuguese-speaking bilingual (Portuguese and English) translators asked to translate the revised version of the original instrument into the English language. The new versions were denominated T1 and T2. Neither translator had prior knowledge of the questionnaire. From these two versions and an analysis performed by the researchers and translators, a single synthesis version was obtained—denominated T3.

The synthesis version of the scale in Brazilian Portuguese (T3) was then backtranslated from Brazilian Portuguese into English by two other bilingual translators with no prior knowledge of the questionnaire. The backtranslated versions were denominated BT1 and BT2. As occurred in the translation step, the researchers and translators created the synthesis version of the backtranslations, resulting in a single version denominated BT3, which was then analyzed by an expert panel.

Nine judges were selected through non-probabilistic convenience sampling based on an analysis of their respective Curriculum Lattes following the criteria cited above to compose the expert panel. All had clinical experience and publications in the field of the disease studied. Women accounted for the majority of the panel (77.7%), which was composed of four endocrinologists, a general practitioner, and four nurses—all with doctoral degrees; 44.4% were associate professors and 77.7% had graduated more than 20 years earlier. A total of 68.6% had more than 20 years of experience and 88.8% had papers published on quality of life and/or diabetes. In terms of expertise, 68.6% reported having experience in the development of scales and 100% reported having experience in psychometrics.

Each of the 45 items on the QSD-R and the 11 items directed at the sociodemographic characterization was analyzed individually by each of the experts and had the following average scores related to the overall CVI: semantic = 0.97; conceptual = 0.98; and cultural = 0.98.

The global average for the CVI considering all categories analyzed was 0.98, which was acceptable, as the recommended value was ≥0.80. The results demonstrate that the items that compose the QSD-R translated and adapted to Brazilian Portuguese have adequate representativeness.

In addition to the majority of items on the QSD-R being considered adequate in the version translated and adapted to Brazilian Portuguese (CVI ≥ 0.80), 88.8% of the items in the semantic category, 93% in the cultural category, and 95% in the conceptual category had CVIs of 1.0. The items that did not achieve the maximum CVI had values of 0.77 and 0.88. These items and the others that were altered are underlined and highlighted in Table 1.

The expert panel made changes to 18 items of the version of the scale translated into Brazilian Portuguese: 1, 2, 3, 5, 6, 7, 9, 10, 13, 14, 15, 17, 18, 23, 24, 34, 37, and 45. The changes mainly consisted of corrections of verbal concordance, the replacement of terms with synonyms, and the restructuring of the item to facilitate understanding. The items that had CVIs of 0.77 and 0.88 in one or two categories were Item 1 (semantic and cultural); Items 10, 13, 18, and 37 (semantic); Item 17 (cultural and conceptual); and Item 28 (conceptual). However, the expert panel did not alter the wording of Item 28.

Items 7 and 9 were the only ones to obtain a CVI of 0.77 or 0.88 in all categories (semantic, cultural, and conceptual). Both items addressed medical therapy and the doctor–patient relationship. The expert panel found it necessary to reword the items to facilitate understanding on the part of respondents. Item 9 (“It bothers me that, whatever I do, I have to take my therapeutic equipment with me”) underwent the most changes. The original translated version was “It bothers me to have to take my “Diabetes kit” with me for anything that I do” and was changed to “It bothers me to have to take my medications and materials for the treatment and control of diabetes for anything that I do”. From the viewpoint of the experts, this item obtained a CVI of 0.77 in all categories (semantic, cultural, and conceptual).

As no item was considered inadequate based on the CVI, none of the items on the original instrument or the translated version used in the pre-test (T3) were excluded. The pre-test version of the QSD-R was administered to 30 patients diagnosed with diabetes. The mean age of the participants was 60.90 ± 16.29 years. A total of 24 (80%) were women and 20 (66.6%) were married or lived with a partner. Fifteen (50%) had a complete high school education and twelve (40%) lived in a home with up to two residents. Concerning the household monthly income, eleven (36.6%) had an income of USD 201 to USD 603 and another eleven (36.6%) had more than USD 2000. Seventeen (56.6%) had been in treatment longer than 10 years.

At this stage, two items merited attention. Item 2 (I have to plan my free time because of my diabetes), translated as “Preciso planejar meu tempo livre por causa da minha diabetes” [I need to plan my free time because of my diabetes], did not require any alteration beyond the removal of the pronoun from minha diabetes (my diabetes), as only one respondent reported not understanding the item. Item 8 (At times I can’t help worrying that I will develop complications later in life) was translated as “Às vezes não consigo deixar de me preocupar com as possíveis complicações da diabetes na minha vida” [Sometimes I can’t keep from worrying about the possible complications of diabetes in my life] was rewritten as “Às vezes me preocupo com as possíveis complicações da diabetes na minha vida” [Sometimes I worry about the complications of diabetes in my life] to facilitate the understanding of the respondents who will effectively participate in the subsequent phases of this study.

Twenty-one participants (70%) reported not being dependent on daily insulin. Although the participants could have relied on the assistance of the researcher for any questions they might have, 100% answered the items individually without the need for any explanations from the researcher. The participants completely understood the vast majority of items on the QSD-R (95.5%).

## 4. Discussion

Formal and objective data collection instruments are scarce for scientific studies across various fields of knowledge in Brazil. As a result, there has been a growing reliance on international instruments and a notable trend of studies focused on translation and cross-cultural adaptation within the country [25].

The present study used the systematic method described in the literature to increase effectiveness. The translation and cross-cultural adaptation process was performed in a discerning manner, considering the close relationship between the CVI and its continuity in future validation studies and the assessment of psychometric properties. Validating pre-existing instruments is a cost-effective and less complicated approach compared to developing new ones. This approach is particularly useful in assessing population patterns, especially in primary experiences that are often international. The constructs used in these experiences produce consistent analysis results when applied in similar scenarios [25,26,27,28].

In this process, the expert panel analyzed the translated and synthesized versions of the QSD-R. This step is important for the inference of specialists with expertise in the fields involved and constitutes the first step in cross-cultural adaptation, followed by the pre-test with the target population. The assessment of the questionnaire by the expert panel was fundamental to obtaining cross-cultural equivalence. The panel was composed of healthcare providers who read the translated version of the instrument and answered a questionnaire expressing their opinions. This step is often omitted but substantially contributes to the understanding of the instrument by respondents [29,30].

In the present study, the experts made suggestions for the correction of spelling and the inclusion of words to nine items. For Items 1, 10, and 34, the suggestion was the inclusion of words to clarify the meaning of the statement. These suggestions were very pertinent since in a country like Brazil, the incidence of diabetic patients with a low level of education is high, as shown by a study carried out in basic health units in the interior of the State of São Paulo, in which the average was 4.54 years of schooling. It is known that a low level of education can favor non-adherence to treatment due to difficulty in understanding, thus increasing health risks and limiting access to information [31].

On the other hand, for other items, the removal of words was suggested. Simple pronouns were excluded from Items 2, 13, and 18, without altering the meaning of the sentences and the CVIs, which varied between 0.77 and 0.88. Only the criteria semantics that aim to assess the level of understanding and acceptance of the terms, the relevance of the items, the existence of any difficulties, and the possible need to adapt the items were altered [32].

Furthermore, important considerations were suggested by the experts for Item 17 (“Sofro com suor excessivo” [I suffer from excessive sweating]). One of the experts (E7) brought up the subjectivity of the item, stating that it would not be adequately interpreted, as Brazil is a tropical country with many hot days. However, diabetes is a disease characterized by the destruction of β cells with deficient insulin secretion, chronic hyperglycemia, and vascular complications compromising the peripheral autonomic nervous system. Therefore, patients with diabetes are more prone to thermoregulatory disorders and sweat gland dysfunction [12,33].

Hoeldtke et al. [34] conducted a longitudinal study comparing individuals with and without diabetes to characterize the presence of sweat gland dysfunction as an early sign of peripheral neural disorder and the quality of the habitual control of the blood sugar level. The authors found a relative increase in the sweating rate (SR) of the forearm and a reduction in the lower limbs, which resulted in an important change in the arm/feet SR ratio in diabetics without adequate blood sugar control. Thus, after a discussion with the expert panel on Item 17, which had a CVI between 0.88 and 1.0, the decision was made to maintain the item due to its agreement with the international literature.

Another important suggestion was to rewrite Items 7, 9, and 37 to facilitate the respondent’s understanding. Such items refer to information provided by doctors, materials for checking capillary blood glucose levels, and how to behave with the diet at parties and restaurants. As these items are specific to the treatment and monitoring of the disease, it was necessary to adapt the nomenclature used to facilitate the understanding of patients who use oral medications, as well as those who use injectable insulin to treat the disease. For these items, the CVI ranged from 0.88 to 1.0, except for Item 9, which had a CVI of 0.77 in the semantic, cultural, and conceptual categories, demonstrating the need for the alteration.

These changes occurred due to the translation process into a language very different from the original, which is English. It is expected that these changes will be proposed with the intention of improving the understanding of the items by diabetic patients. From the translation to the pre-test, the QSD-R remained similar to the original instrument and maintained the same number of items (45 items addressing the daily life of individuals who suffer from diabetes) and the same domains (leisure time; depression and fear of the future; hypoglycemia; self-medication and diets; physical complaints; work; partner; and doctor–patient relationship) plus 11 items addressing sociodemographic characteristics.

It is important to keep in mind that selecting an instrument that was designed in a different language, context, and culture from where it will be used is just the first step. There is a process that needs to be followed to ensure that the instrument is reliable, valid, and effective for use in another culture [25].

We were unable to find any prior studies in the literature that validated and translated the same instrument, which makes it difficult to compare our results with those of others. To ensure the validity of our study, we plan to continue testing the instrument and conduct content validation in a statistically significant sample shortly. The systematic use of the guidelines guided the translation process from preparation to completion of the translation into Brazilian Portuguese. The analysis carried out by the judges in this stage of cultural adaptation ensures the content validity of the instrument. Furthermore, carrying out the pre-test was essential to ensure that the text was understood by the patients. Despite the country having other instruments designed to evaluate the self-care process, treatment management, aspects of life, and the routine of diabetic patients [31], only two have similar profiles, such as the translation of the Diabetes Distress Scale (DDS) instrument), but it has a reduced version of 17 items specific to emotional stress related to diabetes, which would limit the interpretation of the data [32], and the translation of the Type 1 Diabetes Scale (T1DDS), which assesses the emotional suffering of patients with diabetes, for patients with type 1 diabetes [33]. For this reason, the translation and adaptation of this instrument become an important ally for clinical practice, as it covers a greater number of issues related to emotional stress, behavior, and relationships with the disease, without discriminating the type of diabetes.

Study limitations

Participants had a low level of education, which may interfere with understanding the instrument’s items. Another possible limitation refers to the fact that a pilot study was not carried out, although it is not required by the literature adopted [20].

## 5. Conclusions

The translation and cross-cultural adaptation process of the Questionnaire on Stress in Diabetic Patients (QSD-R) showed good linguistics and content validity according to the specialists, revealing its potential for use in clinical practice and future research.

To conclude its validation process, the instrument is ready for the next step, a psychometric assessment of its properties, using the Classical Test Theory.

In this sense, the QSD-R is a promising useful tool for health professionals and researchers to assess stress in diabetic patients considering that DM imposes a considerable economic and social burden on patients and families. However, additional steps are still necessary for its complete validation before implementing its use.

## Figures and Tables

**Table 1:** Items on the version of the QSD-R translated into Brazilian Portuguese altered by the expert panel. Botucatu, SP, Brazil, 2023.

Items		Description	CVI *		
			Semantic	Cultural	Conceptual
TITLE	Original	Questionnaire on stress in diabetic patients (QSD-R).			
	Translation	Questionário sobre estresse em pacientes diabéticos (QSD-R).			
	Result of Expert Panel	Questionário sobre estresse em pacientes diabéticos (QSD-R).	1.0000	1.0000	1.0000
ITEM 1	Original	My diabetes makes me give up tasty foods.			
	Translation	Minha diabetes me faz desistir de experimentar comidas.			
	Result of Expert Panel	Minha diabetes me faz desistir de experimentar comidas saborosas.	0.8889	0.8889	1.0000
ITEM 2	Original	I have to plan my free time because of my diabetes.			
	Translation	Preciso planejar meu tempo livre por causa da minha diabetes.			
	Result of Expert Panel	Preciso planejar meu tempo livre por causa da diabetes.	1.0000	1.0000	1.0000
ITEM 3	Original	I am worried about my spouse/partner.			
	Translation	Estou preocupo com o meu(minha) parceiro(a).			
	Result of Expert Panel	Me preocupo com o meu(minha) parceiro(a).	1.0000	1.0000	1.0000
ITEM 5	Original	If I do not stick to the prescribed treatment, I feel guilty when I see my doctor.			
	Translation	Se não sigo o tratamento prescrito, sinto-me culpado(a) quando vejo meu médico.			
	Result of Expert Panel	Se não sigo o tratamento prescrito, sinto-me culpado(a) quando vou ao meu médico.	1.0000	1.0000	1.0000
ITEM 6	Original	I suffer from “wind”.			
	Translation	Sofro com meus gases intestinais.			
	Result of Expert Panel	Sofro com gases intestinais.	1.0000	1.0000	1.0000
ITEM 7	Original	Different doctors give me different information regarding my diabetes.			
	Translation	Tenho recebido informações diferentes a respeito da minha diabetes vindas de outros médicos.			
	Result of Expert Panel	Tenho recebido informações médicas diferentes a respeito da minha diabetes.	0.8889	0.7778	0.8889
ITEM 9	Original	It bothers me that, whatever I do, I have to take my therapeutic equipment with me.			
	Translation	Incomoda-me ter que levar comigo meu “Kit diabetes” para qualquer coisa que eu faça.			
	Result of Expert Panel	Incomoda-me ter que levar comigo meus medicamentos e materiais para tratamento e controle do diabetes para qualquer coisa que eu faça.	0.7778	0.7778	0.7778
ITEM 10	Original	Often there is not enough food in my diet plan to feel full.			
	Translation	Frequentemente a comida da minha dieta não é suficiente para que eu me sinta satisfeito(a).			
	Result of Expert Panel	Frequentemente a comida descrita na minha lista de dieta não é suficiente para que eu me sinta satisfeito(a).	0.8889	1.0000	1.0000
ITEM 13	Original	Having diabetes means I must eat even if I am not hungry or not having appetite.			
	Translation	Ter diabetes significa que devo comer, mesmo se não estiver com fome ou sem apetite.			
	Result of Expert Panel	Ter diabetes significa que devo comer, mesmo se não estiver com fome ou apetite.	0.7778	1.0000	1.0000
ITEM 14	Original	My physical ability is limited because of my diabetes.			
	Translation	Minha capacidade física é limitada devido a minha diabetes.			
	Result of Expert Panel	Minha capacidade física é limitada devido a diabetes.	1.0000	1.0000	1.0000
ITEM 15	Original	I feel less attractive to others since I developed diabetes.			
	Translation	Sinto-me menos atraente para os outros, desde que descobri a minha diabetes.			
	Result of Expert Panel	Sinto-me menos atraente para os outros, desde que descobri ter diabetes.	1.0000	1.0000	1.0000
ITEM 17	Original	I suffer from excessive sweating.			
	Translation	Eu sofro com meu suor excessivo.			
	Result of Expert Panel	Sofro com suor excessivo.	1.0000	0.8889	0.8889
ITEM 18	Original	Traveling has become complicated and awkward because of my diabetes.			
	Translation	Viajar se tornou complicado e incômodo devido a minha diabetes.			
	Result of Expert Panel	Viajar se tornou complicado e incômodo devido ter diabetes.	0.8889	1.0000	1.0000
ITEM 23	Original	I feel I am insufficiently informed about my diabetes.			
	Translation	Sinto que não sou suficientemente informado(a) sobre a minha diabetes.			
	Result of Expert Panel	Sinto que não sou suficientemente informado(a) sobre a diabetes.	1.0000	1.0000	1.0000
ITEM 24	Original	I have had less sex since the onset of my diabetes.			
	Translation	Tenho praticado menos relações sexuais desde que descobri minha diabetes.			
	Result of Expert Panel	Tenho praticado menos relações sexuais desde que descobri ter diabetes.	1.0000	1.0000	1.0000
ITEM 34	Original	I suffer from intense mood swings			
	Translation	Sofro de intensa mudança de humor.			
	Result of Expert Panel	Sofro de intensas mudanças de humor.	1.0000	1.0000	1.0000
ITEM 37	Original	It is difficult for me to have mention my diet on parties or in restaurants.			
	Translation	É difícil para mim manter minha dieta em festas e restaurantes.			
	Result of Expert Panel	Tenho dificuldade para manter minha dieta em festas e restaurantes.	0.8889	1.0000	1.0000
ITEM 45	Original	At times I worry that my children may also get diabetes.			
	Translation	Às vezes eu me preocupo com o fato dos meus filhos também terem diabetes.			
	Result of Expert Panel	Às vezes, me preocupo com o fato de que meus filhos também possam ter diabetes.	1.0000	0.8889	1.0000

* CVI (content validation index). Footnote: A minimum CVI of 0.80 was established for an item to be considered adequate.

## Data Availability

The data that support the findings of this study are available upon request from the corresponding author. The data are not publicly available due to restrictions, e.g., they contain information that compromises the privacy of research participants. All listed authors meet the authorship criteria and all authors agree with the content of the manuscript.
